# Blockade of the JNK Signalling as a Rational Therapeutic Approach to Modulate the Early and Late Steps of the Inflammatory Cascade in Polymicrobial Sepsis

**DOI:** 10.1155/2015/591572

**Published:** 2015-03-22

**Authors:** Gabriele Pizzino, Alessandra Bitto, Giovanni Pallio, Natasha Irrera, Federica Galfo, Monica Interdonato, Anna Mecchio, Filippo De Luca, Letteria Minutoli, Francesco Squadrito, Domenica Altavilla

**Affiliations:** ^1^Department of Clinical and Experimental Medicine, University of Messina, Messina, Italy; ^2^Department of Paediatric, Gynaecological, Microbiological and Biomedical Sciences, University of Messina, Messina, Italy; ^3^Section of Pharmacology, Department of Clinical and Experimental Medicine, Torre Biologica 5th Floor, c/o AOU Policlinico G. Martino, Via C. Valeria Gazzi, 98125 Messina, Italy

## Abstract

Cecal ligation and puncture (CLP) is an experimental polymicrobial sepsis induced systemic inflammation that leads to acute organ failure. Aim of our study was to evaluate the effects of SP600125, a specific c-Jun NH_2_-terminal kinase (JNK) inhibitor, to modulate the early and late steps of the inflammatory cascade in a murine model of CLP-induced sepsis. CB57BL/6J mice were subjected to CLP or sham operation. Animals were randomized to receive either SP600125 (15 mg/kg) or its vehicle intraperitoneally 1 hour after surgery and repeat treatment every 24 hours. To evaluate survival, a group of animals was monitored every 24 hours for 120 hours. Two other animals were sacrificed 4 or 18 hours after surgical procedures; lung and liver samples were collected for biomolecular and histopathologic analysis. The expression of p-JNK, p-ERK, TNF-*α*, HMGB-1, NF-*κ*B, Ras, Rho, Caspase 3, Bcl-2, and Bax was evaluated in lung and liver samples; SP600125 improved survival, reduced CLP induced activation of JNK, NF-*κ*B, TNF-*α*, and HMGB-1, inhibited proapoptotic pathway, preserved Bcl-2 expression, and reduced histologic damage in both lung and liver of septic mice. SP600125 protects against CLP induced sepsis by blocking JNK signalling; therefore, it can be considered a therapeutic approach in human sepsis.

## 1. Introduction

Sepsis and systemic inflammatory response syndrome (SIRS) are systemic reactions to different inflammatory stimuli such as infection, burns, and trauma [1]. A strict regulation of the inflammatory process is necessary in order to mantain a correct balance between protective or tissue-damaging inflammatory response. If the inflammatory reaction becomes unregulated, systemic and excessive activation of innate immunity results in SIRS or sepsis.

The incidence of sepsis in North America is of 3 cases per 1000 persons, with an estimated increase rate of 1.5% per year. The mortality rate associated with sepsis (40–60%) is so high as to make sepsis the leading cause of death in noncoronary intensive units and one of the major burdens for the healthcare systems throughout the world [2].

Although it has been proposed more than 30 years ago, as experimental model for sepsis induction [3], the CLP model has been considered to be the gold-standard model of sepsis [4]. Sepsis, together with hypotension, acute respiratory distress syndrome (ARDS), hepatic failure, disseminated intravascular coagulation, and organ dysfunction, is associated with a poor prognosis. These alterations occur first in the lung and then in the liver [5].

Bacterial proliferation, endotoxin production, and exotoxin are able to induce an overexpression of pro-inflammatory madiators by macrophages, monocytes, endothelial cells, and neutrophils, thus leading to tissue injuries and organs failure [5, 6].

The NF-*κ*B transcription factor system is known to control the expression of a number of genes involved in the innate immune response of the body against infection and inflammation. Genes responsible for immunoreceptors, cytokines, chemokines, and apoptosis are all modulated by this important family of transcription factors [7]. NF-*κ*B activity is reported to be impaired in chronic inflammation [8]. Recently, we showed that inhibition of NF-*κ*B succeeded in maintaining the balance between pro- and anti-inflammatory cytokines in vivo in a model of polymicrobial sepsis [6]. Phosphorylation of NF-*κ*B and thus transcription of proinflammatory mediators are promoted by the activation of various mitogen-activated protein kinases (MAPKs). MAPKs, such as ERK1/2 and JNK, in turn are activated by bacterial products, cytokines, and chemokines [6–9]. Indeed, JNK is a crucial mediator involved in the activation of proinflammatory cytokines and apoptosis in different cells [9–11]. During septic shock, proinflammatory cytokines such as TNF-*α*, IL-1*β*, and IL-6 are dramatically increased to block the infection and tissue damage [6, 12–15]. Other late mediators of inflammation such as HMGB-1 have been involved in septic shock. In fact, it has been showed that suppressing the HMGB-1 activity exert positive effects in experimental sepsis [16].

Previous in vitro experiments demonstrated that SP600125 acts as MAPKs inhibitor, exhibiting a greater selectivity for all the 3 isoforms of JNK (JNK-1, JNK-2, and JNK-3) rather than for the other kinases; indeed, SP600125 inhibit JNK at a lower concentration then those required to inhibit ERK and p38 (IC50 0.04 *μ*M vs > 10 *μ*M), as previously reported [16]. Furthermore, the dose of 15 mg/kg was described as being able to block the expression of TNF-*α* in a murine model of endotoxin-induced inflammation [16]. Moreover, Bennett et al. showed that the JNK inhibitor significantly reduces the inflammatory response in a model of peritonitis induced lung damage [16, 17]. In light of these considerations, we hypothesized that inhibition of JNK signalling might improve systemic sepsis.

Therefore, aim of our study was to investigate the efficacy and the molecular mechanism of SP600125 in this murine model of polymicrobial sepsis.

## 2. Materials and Methods

### 2.1. Animals, Experimental Procedure, and Treatments

All procedures complied with the standards for the care and use of animal subjects, as stated in the Guide for the Care and Use of Laboratory Animals, and were approved by the Committee on Animal Health and Care of Messina University. The 5-week-old male C57BL/6J mice (Charles River, Calco, LC, Italy), used for this study, had free access to a standard diet and tap water.

They were maintained on a 12-hour light/dark cycle at 21°C.

Cecal ligation and puncture (CLP) was performed in C57BL/6J mice as previously described [18].

The animals (*n* = 35) were randomized in three groups, respectively, Sham (*n* = 7), CLP (*n* = 14), and CLP + SP600125 (*n* = 14); moreover, both CLP and CLP + SP600125 groups were further parted in two other subgroups of seven animals each and sacrificed, respectively, 4 h and 18 h after the treatment. Additionally, 40 animals were also randomized in Sham (*n* = 10), CLP (*n* = 15), and CLP + SP600125 (*n* = 15) and monitored for 120 hours for mortality assessment.

Particularly, mice were anesthetized with ether, and a midline incision was made below the diaphragm to expose the cecum. The cecum was ligated at the colon juncture with a 4-0 silk ligature suture without interrupting intestinal continuity. The cecum was punctured once with a 22-gauge needle. The cecum was returned to the abdomen, and the incision was closed in layers with a 4-0 silk ligature suture. After the procedure, the animals were fluid-resuscitated with sterile saline (1 mL) injected subcutaneously (sc). Sham controls were subjected to the same procedures as were those with CLP without ligation and puncture of the cecum. Shams were treated with SP600125 or vehicle. Animals were randomised to receive either SP600125 (15 mg/kg i.p.) or its vehicle (1 mL/kg of a 10% DMSO/NaCl solution) 1 hour after CLP procedure.

### 2.2. Sample Collection

Samples of liver and lung were collected at both time points (4 h and 18 h) to perform the molecular analysis. At 18 h were also collected specimens of the same tissues to perform histopathologic evaluation.

### 2.3. Isolation of Total Proteins and Western Blot Analysis

After removal, samples of lung and liver were homogenized in 1 mL lysis buffer (25 mM Tris/HCl, pH 7.4, 1.0 mM ethylene glycol tetraacetic acid, 1.0 mM ethylenediamine tetraacetic acid, 0.5 mM phenylmethyl sulfonylfluoride, 10 *μ*g/mL aprotinin, 10 *μ*g/mL leupeptin, 10 *μ*g/mL pepstatin A, and 10 *μ*L/mL NP-40). The homogenate was subjected to centrifugation at 15.000 rpm for 15 minutes. The concentration of total proteins was determined by using the Bio-Rad protein-assay kit (Milan, Italy). The supernatant was collected, mixed with Laemmli sample buffer, and stored at −20°C until analysis.

Western blot analysis was carried out in lung and liver samples to determine p-JNK (Thr183 and Tyr185), p-ERK1/2 (Thr202 and Tyr204), p-NF-*κ*B p65 (Ser536), TNF-*α*, BAX, Bcl-2, HMGB-1, Rho, and Ras levels, as previously described [18]. Equal loading of protein was determined on stripped blots with *β*-actin. Primary antibodies were purchased from Cell Signaling Technologies (p-NF-*κ*B, p-ERK1/2, p-JNK, *β*-actin; Danvers, MA, USA), Abcam (Cambridge, MA, USA; HMGB-1, Rho and Ras), Bio-vision (Milpitas, CA, USA; BAX and Bcl-2), and Millipore (Billerica, MA, USA; TNF-*α*). Secondary, peroxidase conjugated, antibodies were obtained from Thermo Fisher Scientific (Waltham, MA, USA). The protein signals were evidenced by the Enhanced Chemiluminescence (ECL) system and quantified by scanning densitometry by using a bio-image analysis system (Bio-Profil Celbio, Milan, Italy). Results were expressed as integrated intensity compared with those of control normal animals measured within the same batch.

### 2.4. Histological Evaluation

For light microscopy, lung and liver tissues were rapidly removed and fixed in 10% buffered formalin. Subsequently, they were embedded in paraffin, cut, and stained with hematoxylin and eosin (H&E). Assessment of tissue changes was carried out by an experienced pathologist who was blinded to the treatments. The histological study of liver sections was based on the following parameters: infiltration of inflammatory cells, steatosis, necrosis, and ballooning degeneration. The parameters considered for scoring lung damage were infiltration of inflammatory cells, vascular congestion, and interstitial edema. All parameters were evaluated by the following score scale of values: 0, absent; 1, mild; 2, moderate; and 3, severe.

## 3. Results

### 3.1. Effects of SP600125 on CLP Induced Mortality

**Figure 1:**
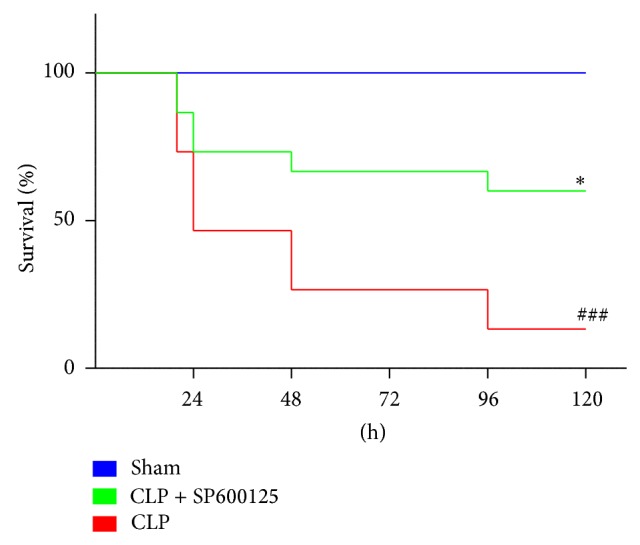


In order to assess the impact of treatment on sepsis-induced mortality, C57BL/6 mice subjected to CLP or sham operation were treated 1 hour after the surgical procedures with SP600125 (15 mg/kg/i.p.) or vehicle. The treatment was repeated every 24 hrs. All the CLP animals were fluid resuscitated* via* administration of sterile 0.9% NaCl saline solution (1 mL/mouse). Animal survival was monitored for up to 120 hours. CLP-induced sepsis in mice produced a significantly higher mortality compared with sham animals (Figure 1). SP600125 administration was able to increase the survival rate in treated animals and reduced mortality in CLP mice (Figure 1).

### 3.2. Effects of SP600125 Treatment on Early p-ERK1/2 and p-JNK Expression

**Figure 2:**
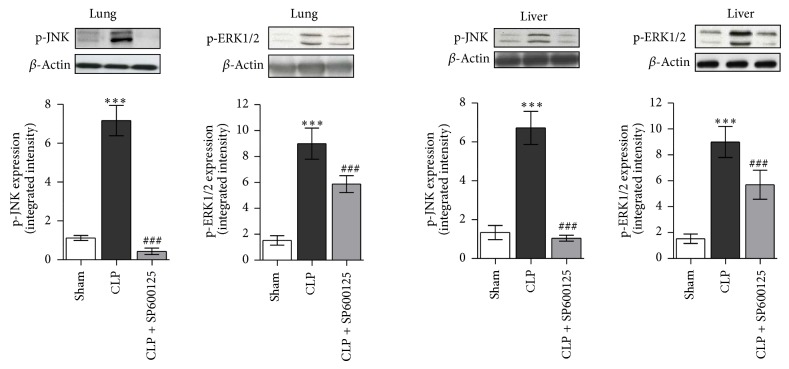


In order to evaluate the effectiveness of MAPKs blockade, we assessed the levels of both and p-JNK in lung and liver 4 hrs after the surgical procedures. As shown in Figure 2, CLP determined an activation of both ERK1/2 and JNK signalling, resulting in a strong increase in phosphorilation of both the proteins in lung and liver of CLP mice. This confirms that MAPKs signalling is an early event in the inflammatory cascade during polymicrobial sepsis. Treatment with SP600125 prevented the phosphorilation and the activation of both ERK1/2 and JNK in both lung and liver when compared with untreated CLP animals (Figure 2). Indeed the inhibitory effect on p-JNK was greater than that on p-ERK, thus confirming that SP600125 is more specific inhibitor of JNK.

### 3.3. Effects of SP600125 Treatment on Early NF-*κ*B and Caspase 3 Expression

NF-*κ*B, one of the main transcriptional factors involved in inflammatory and immune response, is known to be activated by MAPKs; when deregulated and/or robustly activated, it also primes apoptotic cell death. Therefore, we evaluated the levels of p-NF*κ*B to assess the successful blocking of either MAPKs signalling or the extrinsic apoptotic pathway investigated by the means of caspase 3.

**Figure 3:**
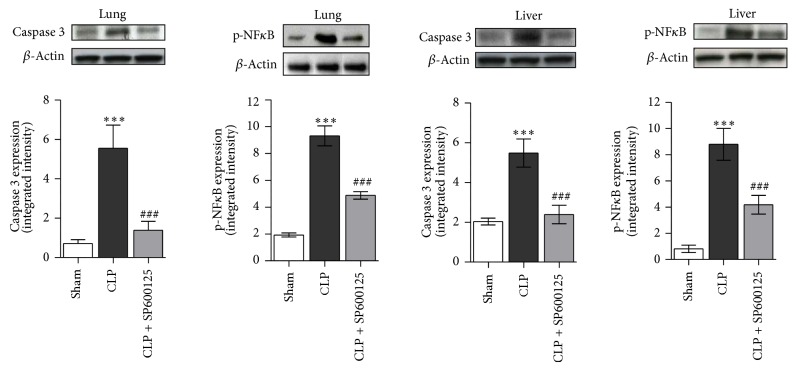


Our results clearly showed (Figure 3) that CLP significantly increases the p-NF*κ*B levels in lung and liver samples, 4 hrs after the surgical procedure, confirming the role of this transcription factor in the early events of the inflammatory cascade. By contrast SP600125 caused, through the inhibition on ERK1/2 and JNK proteins, a significant reduction in NF*κ*B phosphorilation in both lung and liver (Figure 3). In addition the treatment produced a significant reduction in the expression of caspase 3 protein in lung and liver, thus preventing the activation of the apoptotic signalling (Figure 3).

### 3.4. Effects of SP600125 Treatment on Early RHO and RAS Expression

**Figure 4:**
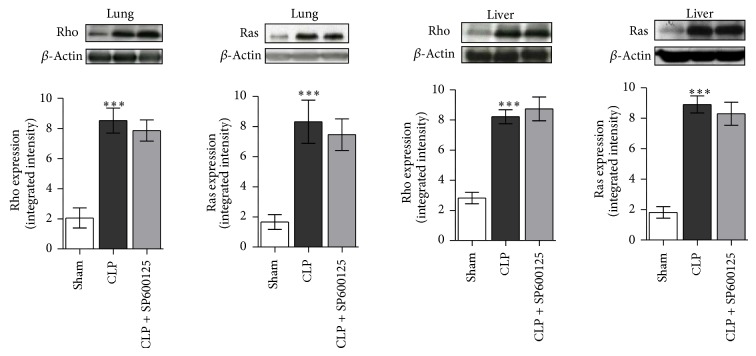


The early signalling culminating in MAPKs activation involves the priming of the Rho and Ras protein. Rho and Ras proteins were overexpressed in CLP animals compared with sham mice (Figure 4) 4 hrs after the surgical procedures. Treatment with SP600125 did not affect Rho and Ras overexpression (Figure 4), thus confirming the specificity of SP600125 on MAPKs.

### 3.5. Effects of SP600125 Treatment on Late TNF-*α* and HMGB-1 Expression

**Figure 5:**
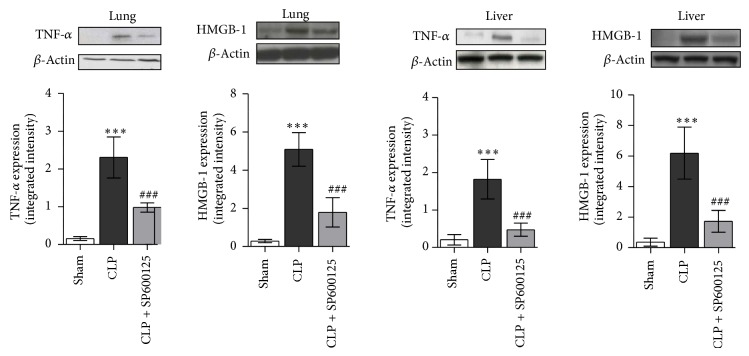


TNF-*α* plays a key role in CLP-induced sepsis; therefore we evaluated the expression of this proinflammatory cytokine 18 hours after the CLP procedure. TNF-*α* expression was significantly enhanced in the lung and liver of CLP animals treated with vehicle compared with sham ones (Figure 5). SP600125 treatment significantly reduced TNF-*α* levels in the lung and liver of CLP mice (Figure 5). Furthermore, considering the proapoptotic role of HMGB-1, we measured this late cytokine in both lung and liver tissue 18 h after the CLP procedure. Administration of SP600125 reduces the expression of this late cytokine protein in both lung and liver tissue of CLP animals (Figure 5).

### 3.6. Effects of SP600125 Treatment on the Late BAX and BCL-2 Expression

**Figure 6:**
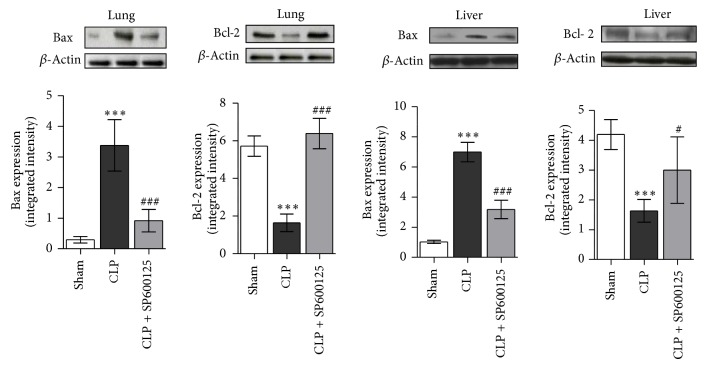


Eighteen hours after CLP, BAX levels were significantly enhanced while Bcl-2 protein significantly reduced in both lung and liver (Figure 6). Treatment with SP600125 decreased BAX expression and enhanced BCL-2, thus suggesting the specific JNK inhibitor blunts the activation of proapoptotic signal in polymicrobial sepsis.

### 3.7. Effects of SP600125 on Histopathology Features

**Figure 7:**
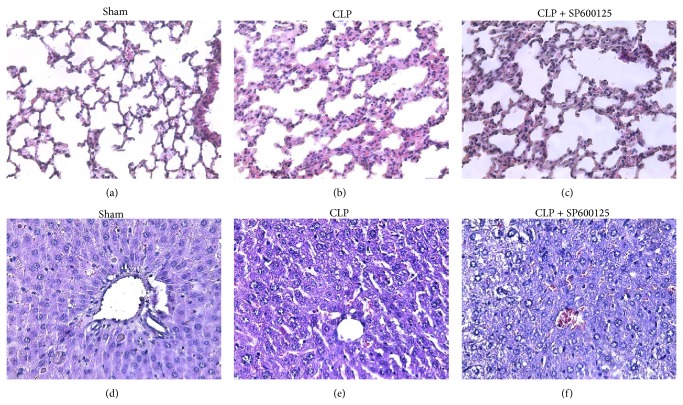


As shown in Figure 7 and in Table 1, CLP caused significant changes in the architecture of both lung and liver. In the lung of CLP animals an increased inflammatory infiltrate was observed as well as a consistent augmentation of both the oedematous and hemorrhagic areas when compared with sham animals (Figures 7(a) and 7(b) and Table 1). By contrast specimens from CLP treated animals showed reduced edema, inflammation, and vascular congestion (Figure 7(c) and Table 1).

CLP caused a liver damage characterized by a diffuse inflammatory infiltrate, relevant steatosis, necrosis, and also ballooning degenerated areas (Figures 7(e) and 7(d) and Table 1). SP600125 caused a reduction in steatosis and necrosis (Figures 7(f) and 7(e) and Table 1) and decreased the damage induced by CLP.

## 4. Discussion

The inflammatory cascade primed by CLP-induced polymicrobial sepsis is known to be correlated with an overwhelmed production and secretion of proinflammatory cytokines that could lead to several pathological consequences, such as accumulation of leukocytes, apoptotic cell death, and necrosis, finally causing multiple organ failure.

SP600125 has been shown to inhibit the MAP-kinase JNK and to partially antagonize ERK1/2, by a not specific and indirect action [16]. Both kinases are majors players involved in mediating and transferring the early inflammatory stimulus from cell membrane to the nucleus [9, 10]. Therefore the blockade of this early step represents a rationale therapeutic approach to limit the pathological inflammatory cascade during septic states.

In this study we showed that the pharmacological inhibition of JNK, induced 1 hour after the CLP procedure* via* a repeated SP600125 administration (every 24 hours), was able to achieve a significant reduction in the inflammatory process and to cause a marked improvement in survival of treated mice compared with untreated animals (Figure 1). This result is of particular interest: in fact in a clinical setting the improvement in the survival may allow creating a therapeutic window in which the underlying antibiotic drugs are more likely to reach the therapeutic efficacy.

Cecum ligation and puncture is an experimental model of polymicrobial sepsis that allows dissecting out the early and late components of the inflammatory cascade. Therefore we used this experimental paradigm to investigate the precise molecular pathways that are targeted by SP600125. Our hypothesis was that the drug may block the early and late steps of the inflammatory cascade by inhibiting JNK kinase: indeed we were able to demonstrate that SP600125 blunts JNK activation in both lung and liver. However CLP also triggered the activation of Rho and RAS proteins, two additional components of the proinflammatory pathway, which might lead in turn to JNK and ERK1/2 phosphorilation, with consequent activation of NF-*κ*B.

To investigate this molecular pathway we studied the effects of SP600125 on these two proteins and we found that the drug did not change the expression of Rho and RAS, thus ruling out the hypothesis of an interference with these two “actors” of the inflammatory cascade.

The net results of the early steps of the molecular cascade are an increased production of TNF-*α* and HMGB-1, two cytokines responsibles, at the initial stage of the inflammatory process, for the increase in leukocytes infiltrate and edema, and at later stage apoptosis and necrosis that may culminate in systemic organ failure.

These complex sequelae of events are evidenced by our experiments. Blocking the up-stream signalling mediated by JNK kinase was able to downregulate the TNF-*α* levels in both lung and liver tissues, thus producing an improvement in the histological features of both these organs as confirmed by the histopathologic evaluations (Figure 7(f)). Moreover, SP600125 treatment caused the same degree of downregulation in the proinflammatory protein HMGB-1 and in the proapoptotic caspase 3 and BAX together with an upregulation of Bcl-2, a well-known antiapoptotic mediator.

These molecular findings paralleled with the histologic evaluations carried out on liver and lung samples, which depict a general restoring of the normal architectural characteristics of both tissues; in fact we observed a regression of inflammatory infiltrate as well as a reduction of both interstitial edema and vascular congestion in the lung. Furthermore a more well-structured parenchyma, with partial reorganization of hepatic lobule architecture, a general reduction of both steatotic and necrotic areas, and a regression of the ballooning degeneration phenomenon were observed in the liver.

In conclusion our findings clearly suggest that the blockade of the JNK mediated signalling may represent an innovative and effective approach in the management of polymicrobial sepsis.

## Figures and Tables

**Table 1:** SP600125 effects on lung histologic damage in CLP mice. ∗∗∗ is for *P* < 0.0001.

	Parameters	Sham	CLP	CLP + SP600125
Lung	Inflammatory infiltrate	0	2.31 ± 0.41	1.28 ± 0.23^***^
	Vascular congestion	0	2 ± 0.18	1.1 ± 0.37^***^
	Interstitial edema	0	2.12 ± 0.53	1.32 ± 0.44^***^

Liver	Inflammatory infiltrate	0	2.1 ± 0.39	1.3 ± 0.33^***^
	Steatosis	0	2.89 ± 0.56	1.56 ± 0.26^***^
	Necrosis	0	2.2 ± 0.47	0.9 ± 0.21^***^
	Ballooning degeneration	0	2.76 ± 0.47	1.58 ± 0.48^***^
